# Sex differences in the brain: a whole body perspective

**DOI:** 10.1186/s13293-015-0032-z

**Published:** 2015-08-15

**Authors:** Geert J. de Vries, Nancy G. Forger

**Affiliations:** Neuroscience Institute, Georgia State University, P.O. Box 5030, Atlanta, GA 30302-5030 USA

**Keywords:** Brain, Adipose tissue, Bladder, Environment, Gut, Immune system, Kidney, Liver, Muscle, Placenta, Sensory system, Sex difference

## Abstract

Most writing on sexual differentiation of the mammalian brain (including our own) considers just two organs: the gonads and the brain. This perspective, which leaves out all other body parts, misleads us in several ways. First, there is accumulating evidence that all organs are sexually differentiated, and that sex differences in peripheral organs affect the brain. We demonstrate this by reviewing examples involving sex differences in muscles, adipose tissue, the liver, immune system, gut, kidneys, bladder, and placenta that affect the nervous system and behavior. The second consequence of ignoring other organs when considering neural sex differences is that we are likely to miss the fact that some brain sex differences develop to compensate for differences in the internal environment (i.e., because male and female brains operate in different bodies, sex differences are required to make output/function more *similar* in the two sexes). We also consider evidence that sex differences in sensory systems cause male and female brains to perceive different information about the world; the two sexes are also *perceived by* the world differently and therefore exposed to differences in experience via treatment by others. Although the topic of sex differences in the brain is often seen as much more emotionally charged than studies of sex differences in other organs, the dichotomy is largely false. By putting the brain firmly back in the body, sex differences in the brain are predictable and can be more completely understood.

## Review

### Introduction

If this were a review about sex differences in skin, bone, kidney, liver, or just about any other peripheral organ, chances are slim that anyone would take offense. Discussing sex differences in the brain puts this paper in a different class altogether and probably for good reasons. We credit our brains for who we are, how we behave, and what we achieve. There seems to be more at stake in believing that brains are different between two groups of people than in believing the same thing about other body parts, which makes the topic of this paper anything but neutral.

In fact, however, sex differences in the nervous system are not really separable from sex differences in other body parts. For example, hormones may affect behavior by acting directly on the brain or more indirectly via a peripheral organ, whose function affects the brain. (Or even more circuitously, such as when hormonal effects on the body change the way individuals are treated and, hence, their experience and the brain.) The whole body is sexually differentiated, and no organ (the brain included) operates in isolation.

As a “thought experiment,” it may be useful to consider a sex-neutral brain hooked up to either a male or female body. Would the brain “know” the sex of the body it was in? How long would this take? Would effects accumulate over time? A few moments reflection will probably lead to the predictions: yes…, not long…, and probably. For starters, there are obvious things like differences in gonadal steroid hormones to which the brain is directly exposed. However, as discussed below, many other factors that affect the brain differ in male versus female bodies based on differences in gene expression, biochemistry, and the structure of peripheral organs.

History justifies a cautious approach when approaching a topic like “brain sex.” About a century ago, sex differences in the brain were linked to presumed differences in intelligence and were used to legitimize a diminished role for women in society [1]. Even though that interpretation has long since been debunked, unsubstantiated claims about the nature and function of neural sex differences continue to be made and such claims may do serious harm [2–4]. But neglecting sex differences in the brain (or any other body part), or politicizing the discussion of their significance, also has its costs [5].

We believe that ignoring sex differences in the brain, however they arise, compromises best practices in biology and medicine, in some cases with substantiated, negative health effects. A sex difference in a physiological process is one of nature’s ways of demonstrating how that process can be modulated. Sex differences in the vulnerability to a disease may similarly reveal factors that are protective in one sex, thereby suggesting strategies to prevent or ameliorate that disease. This is especially true for many neurodevelopmental disorders, where “sex” explains more of the variance than any other known contributing factor [6–10]. In humans, some treatments are known to be more effective in one sex than the other, and optimal drug doses for men and women may differ [11]. We ignore these things at our peril.

Perhaps by viewing sex differences in brain and behavior in the context of the many influences from inside and outside the body, we can see the important topic of neural sex differences in a fresh perspective. In this review, we will discuss research on sex differences in the nervous system and peripheral organs in mammals. We point to several ways that sex differences in the periphery may affect brain function and, finally, touch on how factors outside of the individual may influence the brain.

### There are sex differences in the brain

In the past four decades, many sex differences have been found in brain structure and chemistry [12–15], usually without researchers making spectacular claims as to their significance. Taken together, these findings lead to the inescapable conclusion that male and female brains differ.

The function of some neural sex differences can be understood intuitively. An example that we expand on below is the well-studied sex difference in the number of motoneurons controlling the striated perineal muscles, which mirrors a sex difference in the size of the target muscles themselves. In many cases, however, the functions of neural sex differences are mysterious. We have made the case that although some sex differences may cause differences in function, in other cases sex differences exist to ensure that function is similar in males and females. In other words, some sex differences compensate for physiological differences that if left unchecked may be maladaptive [16]. Both sexes have to eat, drink, breathe, regulate body temperature, and run a host of other homeostatic processes, and nature finds a way to adjust these processes in sex-specific ways. In the end, male brains are in male bodies, and evolution has seen to it that they serve these bodies best, and the same is true for females [17].

Sex differences that perform a compensatory role may become evident when the system is perturbed. A specific example comes from looking at something as seemingly basic as cell death programs in neurons. In response to hypoxia, or other conditions mimicking stroke, neurons die in both sexes of rats and mice. However, the underlying molecular pathways of cell death differ, as becomes clear when pharmacologic or genetic manipulations that inhibit the cell death pathway used by, for example, male cells, only ameliorates the effect of stroke in males [18, 19]. In this case, an intervention reveals a fundamental sex difference.

Another example concerns one of the most pervasive sex differences: the inactivation of one X chromosome in every cell of the body in females. Random X inactivation is perhaps the prime example of a sex difference (in this case, a process that happens in all female cells and no male cells) that exists in order to make the sexes more similar (more or less equalizing the dosage of X chromosome genes). For the most part, nature does a great job in covering up the consequences of this. However, the inactivation of an entire chromosome in each female cell utilizes epigenetic machinery, and the inactivation state must be continually maintained [20, 21]. There is evidence that this affects the expression of autosomal genes [22, 23], presumably because there is a limiting supply of the DNA methyltransferases and histone-modifying enzymes required for the epigenetic changes that underlie the inactivation of an entire chromosome. It is not hard to see how this one event (usually thought of in terms of equalizing males and females) may have ripple effects that result in sex differences elsewhere.

In much the same way that X chromosome inactivation alters the cellular context, sex differences in peripheral systems also contribute to the different “contexts” in which male and female brains operate. One familiar example concerns gonadal steroid hormones in the general circulation. The peripheral blood of males and females contains different levels of steroid hormones in large part due to differences in production by the gonads. But activity in other organs, e.g., hormone metabolism in the liver or rates of excretion by the kidneys, also contributes to measurable differences in circulating steroid levels [24, 25]. To the extent that gonadal steroids in circulation differ, most of us are comfortable expecting an effect on the brain, but few of us think about the role the liver or kidneys have played. Although steroids may be the most familiar example, many other constituents of blood also reach male and female brains in different levels. Identifying the ways the internal “environment” differs when a brain is in a male versus a female body should provide insight into the development, maintenance, and function of sex differences in the brain, including their role in disease. In this review, we will make the case that a full understanding can only be achieved against the perspective of the entire body (and beyond).

### Causes of sex differences in the brain

For mammals, sex is determined at conception. If the fetus has inherited a Y chromosome, it will develop testes. Hormonal products of the testes, mainly testosterone, then induce the male phenotype by early permanent *programming* effects (originally called organizational effects) and later transient *acute* effects, which disappear after withdrawal of the hormones (also called activational effects). In the absence of a Y chromosome, the fetus develops ovaries, and in the absence of male-like levels of testosterone, the female phenotype emerges. The activating effects of ovarian hormones enhance female characteristics at puberty and beyond.

For much of the latter half of the last century, sex chromosomes were not considered to play an important differentiating role, apart from the crucial first step of directing the early development of the gonads. However, not all sex differences could readily be reversed by altering gonadal hormone levels experimentally, and for some sexually dimorphic traits (e.g., plumage in zebra finches and mammary tissue in wallabies), there was overwhelming evidence that sex differences were hormone-independent [26]. More recently, it has become clear that sex chromosomes play a direct role in establishing sex differences throughout the body, including the brain [27, 28]. The development of the four core genotype mouse model, in which sex chromosome status and gonad type are inherited independently, has been decisive in showing that some differences depend on chromosomal constitution (XX versus XY) and are gonad independent [28, 29].

Nonetheless, for the large majority of neural sex differences that have been described, sex differences in gonadal steroid hormones seem to play a dominant role. The most parsimonious explanation for how sex differences in the nervous system develop might therefore be that gonadal hormones act directly on the neural tissues that differentiate. This is certainly the case in some instances, but as described below, direct effects of steroids are just one of several possibilities.

#### Hormones may act on steroid receptors in tissues that differentiate

In early papers on sexual differentiation, it was *de rigueur* to include a description of areas that express receptors for gonadal steroid hormones (androgen and estrogen receptors), the implication being that those were the areas most likely to be the direct targets of the differentiating effects of gonadal hormones. It is indeed a good place to start, and in fact, evidence for direct effects of steroids on the regions that differentiate was found in many cases. For example, specific brain regions were shown to express androgen and estrogen receptors during early development [30–34], and the application of testosterone into such regions defeminized the luteinizing hormone surge in female rats [35, 36] and masculinized juvenile play behavior and ultrasonic vocalizations of female rats and gerbils, respectively [37, 38]. Blocking hormone action by injecting estrogen receptor mRNA antisense oligonucleotides locally within the hypothalamus also prevented some of the defeminizing and masculinizing effects of testosterone treatment of neonatal female rats [39].

More recently, a direct effect on the brain area in question seems to be assumed more often than proved. If an effect is seen and gonadal steroid receptors are expressed in that region, it is unusual for an investigator to go further, even if the gonadal steroid receptors in question have been demonstrated during adulthood, not during the perinatal critical period.

#### Hormones may act on steroid receptors in other brain areas that, in turn, influence differentiating areas

Even when a given brain region expresses the relevant steroid receptors, hormones may act elsewhere to differentiate that area. A good example is the sexually dimorphic projection from the bed nucleus of the stria terminalis (BNST) to the anteroventral periventricular nucleus of the hypothalamus (AVPV). This pathway is ~20-fold more dense in male rats and is dependent on early exposure to testosterone [40]. The BNST abundantly expresses androgen and estrogen receptors, and the nucleus is larger in males, so it might be assumed that hormones act at the BNST to influence the outgrowth of BNST axons. To test this, Simerly and colleagues turned to a co-culture system. They first confirmed that the in vivo sex difference was recapitulated *in vitro*: neurite outgrowth from the BNST was much greater in co-cultures of the BNST and AVPV dissected from developing male rat pups than in co-cultures of the BNST and AVPV of females [41]. However, in a mix-and-match experiment, in which the BNST of males (or testosterone-treated females) was co-cultured with the AVPV of females (or vice versa), it was clear that hormone action at the target site (AVPV) determines the size of the projection from the BNST [41]. This kind of mechanism has been appreciated for many years in steroid-dependent development of the periphery. For example, the effects of testosterone on the developing prostate epithelium and mammary gland rudiments in male rodents (in one case to cause growth and differentiation, and in the other case, destruction) are mediated indirectly, by hormone action on neighboring cells [42, 43].

#### Hormone action on peripheral structures may in turn influence the nervous system

Perhaps nowhere has the “site of action” question for an effect of gonadal steroids on the nervous system been pursued as vigorously as it has for the spinal nucleus of the bulbocavernosus (SNB) of rats, and for that system, the answer lies primarily outside of the nervous system. Motoneurons of the SNB innervate striated muscles in the perineal region including the bulbocavernosus and levator ani. These muscles wrap around the base of the penis and contract during erection and ejaculation. In adulthood, male rats have well-developed bulbocavernosus and levator ani muscles and about 200 SNB motoneurons. Females lack the muscles almost entirely and have only about 50 SNB cells [44]. Although best studied in rats, a similar sex difference is seen in other mammals, including humans [45–49].

The sex difference in the rat SNB is completely dependent on androgens around the time of birth [50, 51]. Moreover, the SNB motoneurons express androgen receptors in adulthood [44], which was exciting because it suggested that a steroid hormone might act on cells within the central nervous system to cause sex differences (a concept with few direct demonstrations at that time). However, it soon became apparent that for the most conspicuous sex difference associated with the SNB—that of motoneuron number—the site of hormone action was unlikely to be the motoneurons themselves. SNB motoneurons do not express the androgen receptor until *after* androgens have determined the fate of these cells [52], and androgens can spare SNB motoneurons that themselves do not express the receptor [53]. Instead, the site of hormone action appeared to be the muscles these neurons innervate [54, 55]. The muscles form in both sexes prenatally but degenerate by apoptosis unless they are exposed to testosterone around birth [56]. Persistence of the muscles is required for SNB survival, perhaps because the muscles produce trophic factors required by the motoneurons [57]. If so, then hormones change something in the body (the perineal muscles) that, in turn, changes something in the central nervous system (motoneuron number in the spinal cord).

Androgen action on striated muscle fibers cannot be the whole explanation for the sex difference in SNB motoneuron number, however, because if androgen receptors are expressed in only striated muscles cells, and no other cell type, this is not sufficient to rescue the SNB system [58]. Other site(s) of hormone action (which could be other cell types in the periphery or in the CNS) presumably also contribute. In adulthood, androgens also act at the target muscle to influence dendritic extent of the motoneurons [59, 60], whereas other actions of testosterone on SNB cells (e.g., control of soma size) appear to be direct [61, 62].

Because it has been studied in such detail, the SNB is a “poster child” for how hormones can act in the periphery to affect the nervous system [63]. For most of the other examples described below, fewer details are known, but evidence suggests that there are a variety of routes and mechanisms by which the periphery can affect the brain.

### Whole body perspective of sexual differentiation of the brain

In the next paragraphs, we discuss additional examples showing that sexual differentiation of organs and tissues other than the nervous system itself eventually affect neural function or morphology. We do not attempt to cover all possible examples but provide several concrete examples involving an array of organ systems.

#### Pelvic viscera

Another consequence of early sexual differentiation is that the pelvic viscera differ in males and females (e.g., seminal vesicles, epididymis, and prostate in males; uterus and fallopian tubes in females). This, perhaps not surprisingly, is accompanied by sex differences in innervation. The viscera receive parasympathetic, sympathetic, and sensory innervation via the pelvic, hypogastric, and pudendal nerves [64], and for all three modalities, sex differences have been described (either in the number of innervating cells or fibers or the pattern of innervation; e.g., [65–67]). For example, sensory innervation of the perineum provided by the pudendal nerve differs in males and females. The cell bodies providing this innervation, which reside in the dorsal root ganglia, are more numerous in male rats than in females [68, 69]. Although details are lacking regarding how this sex difference develops, it may be due to greater neuronal cell death in females, as in the SNB [69].

Perhaps less obvious are sex differences in the innervation of peripheral structures that are present in both sexes, such as the bladder, which receives sympathetic innervation from the hypogastric nerve. When Nadelhaft and McKenna applied a retrograde tracer to the stump of the severed hypogastric nerve of rats, they found almost four times as many labeled preganglionic sympathetic neurons in the spinal cord in males than in females [70]. A similar sex difference was seen in guinea pigs [67]. When one keeps in mind that these sympathetic neurons do not exist in isolation, but themselves receive innervation, the possibilities for cascading effects on ever higher levels of the nervous system are clear.

Brain areas involved in micturition (emptying the bladder) [71] show higher levels of activation in men than in women during conscious control of the pelvic muscles that control urine flow [72]. The bladder has sex differences not just in innervation (above and [73]) but in neurotransmitter receptors [74], structure of the bladder wall [75], and size of the external urethral sphincter muscle [76]. As there are also sex differences in pathophysiology, e.g., women are more likely to show overactive bladder activity coupled with incontinence than are men [77, 78], and boys are more likely to show nocturnal enuresis (bedwetting) than girls [79, 80], full understanding of interactions among sex differences in the brain and body may be required for developing optimal treatments of these conditions in males and females.

#### Adipose tissue

Men and women have differences in adipose tissue distribution. Women tend to accumulate subcutaneous, femoral, and gluteus fat whereas men deposit fat within the abdomen [81]. Adipose tissue is an endocrine-signaling organ, producing a number of peptide and steroid hormones (e.g., leptin, adiponectin, inflammatory cytokines, and estrogens) some of which reach the brain and act on receptors there (see [82–84] for review). Interestingly, these signals may differ, on average, in males and females because of the differences in the metabolic profile of subcutaneous versus visceral fat. For example, the increased visceral fat seen in obese men compared to obese women contributes to elevated inflammatory cytokines in men [85].

Although gonadal steroid hormones are known to play a role in fat deposition, recent research also suggests a gonad-independent role for genes on the sex chromosomes. Mice with two X chromosomes, regardless of whether they have testes or ovaries, have more fat [86]. This effect is most clearly seen in mice of the four core genotype model mentioned above: after removal of the gonads, XX females have twice the adipose tissue as XY females and are much more likely to develop fatty liver [86]. Although a similar experiment cannot be done in humans, abnormalities in sex chromosome numbers as in Klinefelter (XXY males) and Turner syndrome (X0 females) are associated with abnormalities in adipose tissue distribution [87, 88].

Thus, both gonadal steroids and sex chromosomes may influence the brain via effects on adipose tissue. In addition, adipose tissue receives sympathetic innervation, and this innervation differs by sex and fat pad [89]. It is not known whether sympathetic neurons innervating adipose tissue become different in response to their sexually dimorphic targets (in analogy to sex differences in the SNB motoneurons that innervate the bulbocavernosus muscle described earlier) or, conversely, if differential innervation plays a role in causing the sex differences in body fat distribution [90].

#### Liver

The liver secretes steroid-binding proteins and contains enzymes that metabolize circulating gonadal steroids [24, 25, 91, 92]. These enzymes may influence the level of gonadal steroids differently in males and females. To some extent, we have already factored in the action of the liver (albeit, usually without thinking about it) when we measure differences in circulating hormone levels. We may think that differences are caused by different levels of hormone secretion by the gonads, but these levels are the result of local production and metabolisms by various organs, the liver being one of the most important. Moreover, following an acute treatment with the “same” dose of steroid, males and females may be subject to differences in biologically active steroid that reaches the brain due to sex difference in liver function. Different function of the liver would also affect metabolism of environmental estrogens such as phytoestrogens, which would again affect steroid-responsive systems in the brain differently in the two sexes.

Although we do not normally think of the liver or adipose tissue as being very sexually dimorphic organ systems, the expression of 72 % of all genes surveyed in the liver and 68 % of adipose tissue genes differs between male and female mice [93]. These percentages are much larger than one would expect based on the modest sex differences in the overt form and functions of liver and adipose tissue. To gain some perspective, however, the liver supplies the majority of yolk proteins in egg-laying species, and ultrastructural sex differences in the liver are required to accommodate this massive protein production in females [94]. Although the requirement for a sexually dimorphic liver is less obvious in species bearing live young, the maternal liver also synthesizes proteins important for fetal development in mammals [94].

The liver also metabolizes most drugs, leading to sex differences in pharmacodynamics and pharmacokinetics [25]. A good example of a drug with lower clearance in women than in men is the sleep aid, zolpidem (trade name: Ambien) [95]. Sex-specific dose recommendations for this drug were issued in 2013 by the U.S. Food and Drug Administration, 20 years after it came to market [96], and zolpidem is currently the only drug with different recommended doses for men and women. In general, however, women have more adverse drug reactions than men [11, 97], and there are likely many more drugs on the market that should have sex-specific dosing. When the Food and Drug Administration of the U.S.A. reviewed new drug applications between 1995 and 2000, only half included analysis by sex, and of those, 6–7 % showed a large (>40 %) difference in pharmacokinetics between men and women [98].

#### Peripheral immune system

Sex differences in immune activation are observed in response to the same stimulus [99, 100] and may underlie well-established sex biases in immune-related diseases. Women have a higher incidence of most autoimmune diseases [101, 102] and may also have increased responses to tumors and infections. Differences persist when lymphocytes harvested from men and women are stimulated in vitro [103], which is important because it rules out potentially confounding effects of the cells being in a male versus a female body. Differences in immune activation or inflammation in males and females during critical periods of development have been linked to sex-specific effects on brain functions such as learning and memory, locomotion, and emotional regulation [104]. A recent meta-analysis concludes that many of the sex differences in immunological response are attributable to the immune-regulatory effects of sex steroid hormones [100]. However, the sex chromosomes may also play an important role, as illustrated by the next example.

Multiple sclerosis (MS) is a neurodegenerative disease with a whole-body explanation involving multiple peripheral organs and contributions from both gonadal steroids and sex chromosomes. In MS, a patient’s immune system attacks components of the myelin sheaths encircling axons, and the disease is more prevalent in women than in men. Females are also more susceptible in a mouse model of MS (experimental autoimmune encephalomyelitis), and the explanation for this is distributed across body parts. To induce the MS-like disorder, mice receive injections of myelin basic protein and immune-boosting agents near lymph nodes. A sex difference is already found in the initial induction phase of the disease, such that male mice have fewer lymph node immune cells and fewer reactive cells that produce inflammatory cytokines than do females in response to inoculation with the myelin basic protein [105]. If this sex difference in the induction phase is taken out of the equation by transferring myelin basic protein-specific T lymphocytes from females into mice of both sexes, males still show protection to disease onset [106], and one mechanism appears to be increased production of anti-inflammatory cytokines by male spleen cells. Thus, the periphery presents the brains of males and females with different stimuli given the same disease trigger.

Although numerous studies have proposed protective roles for gonadal steroid hormones in MS (reviewed in [107]), and sex steroids are being tested in MS clinical trials, there are also likely to be important direct chromosomal effects. Using the four core genotype mice in the mouse model for MS mentioned above, XY lymph node cells show a smaller immune response in the induction phase regardless of the gonad type of the donor [108]. Interestingly, however, when the sex of immune system cells is held constant (e.g., XX immune system in an XX or XY animal), mice with an XY nervous system show more severe pathology [109]. This may be related to the clinical observation that although MS is more prevalent in woman, men with MS have faster disease progression [110, 111].

Depression, a stress-related disorder, is also more common in women than in men [112, 113]. In most cases, we would look to the brain to explain a difference in susceptibility to depression, but recent findings suggest it could be linked to individual or sex-based differences in activation of the peripheral immune system. An increase in the number of white blood cells is reported in men with major depression [114]. When male mice are subjected to a social stressor, some subsequently exhibit depressive-like behavior, whereas others are resilient. Hodes and colleagues noted that there were significant differences in the peripheral immune response to stress between mice that subsequently were susceptible or resilient. Surprisingly, resilience could be “transplanted”: susceptible mice receiving a bone marrow transplant (and, hence, peripheral immune system) from resilient mice themselves became resilient [115]. This suggests that the peripheral response to a stressor may be an important factor determining the onset of depression. Although this study included only males, previously reported sex differences in the peripheral immune response to stressors suggest that in some cases depression and anxiety disorders may be traced to the periphery.

In addition to effects of the peripheral immune system on the brain, recent work suggests that the brain itself uses elements of immune or inflammatory signaling in the process of sexual differentiation. For example, prostaglandins—best known for their roles in inflammation—are required for masculinization of copulatory behavior and dendritic spine density in the preoptic area of the hypothalamus in rats [116]. Moreover, masculinization can be blocked by neonatally preventing brain prostaglandin synthesis [116] or by inhibiting microglia, the innate immune cells of the brain and producers of prostaglandins [117].

#### Gut

Gene expression in the small intestine and colon differs in male and female mice and does so even before weaning [118, 119]. In addition, adult males and females housed in the same environment and eating the same food exhibit differences in the gut microbiota (the collection of microorganisms living symbiotically in our intestines) [120]. These differences can account for differences in behavior or susceptibility to disease. In a mouse strain that is susceptible to type 1 diabetes, for example, females are more often affected than males, but the sex difference disappears when the mice are raised germ free (i.e., in the absence of a microbiome) [120]. This suggests a role for the microbiota in the sex difference in diabetes susceptibility. To test this more directly, Markle and colleagues transferred the gut microbiota from adult males to juvenile females of the diabetes-susceptible strain. The female recipients’ microbiota, hormone levels, and serum metabolites were all altered, such that they were now diabetes resistant [120]. Thus, the microorganisms that live in us and on us may differ by sex and cause differences in disease.

Recent work also suggests that the microbiota has acute as well as programming effects on the brain and behavior. For example, mice treated with valproic acid on gestational day 11 are well-established animal models for autism. These mice have reduced social interactions and increased markers of gut inflammation, but both changes are found in males only [121]. The same valproic acid treatment also significantly alters the composition of bacteria comprising the gut microbiota, and social behavior correlates with the levels of metabolites (lactate, butyrate, and acetate) generated by these microorganisms [122]. Although quite interesting, this evidence is indirect because many factors in addition to gut microbiota may be affected by gestational exposure to valproic acid. More direct evidence was obtained very recently by showing that germ-free mice were less sociable and did not show the typical preference for investigating novel mice [123]. Colonizing germ-free mice with a microbiota at day 21 only partially normalized social responses in adulthood [123], suggesting that microbiota must be present even earlier for completely normal social behavior. This study also found stronger effects of early exposure to microbiota in males than in females.

The gut microbiota has various ways to exert its influence on the brain. The best substantiated of these is signaling via the vagus nerve, which can transmit information related to local changes in the gut wall to the brain. But gut microbiota can also signal via the bloodstream, e.g., by changing levels of free fatty acids, neurotransmitter precursors, and inflammatory cytokines produced by immune cells in the gut wall in response to specific gut pathogens [124]. A very intriguing possibility is that the microbiota can also influence circulating levels of steroids such as testosterone [120], which would of course interact with programming and acute effects of steroids produced by the gonads.

#### Kidney

The role of the kidney is to remove the waste products of metabolism while also maintaining water balance. Vasopressin, a peptide hormone synthesized in the hypothalamus and released by the posterior pituitary, is crucial in conserving water loss (hence its other name—antidiuretic hormone). The vasopressin V_2_ receptor (V_2_R) mediates the antidiuretic effect of vasopressin on the kidney, and female mice express about twice as much V_2_R mRNA and more than twice as much V_2_R protein than do males [125]. This difference probably results from the location of the V_2_R gene on the X chromosome. Although most X chromosomal genes are expressed at comparable levels in males and females due to random X inactivation, this inactivation is not complete. In humans, up to 15 % of X chromosomal genes escape inactivation to varying degrees [126], and of these, the V_2_R gene has among the highest levels of escape [126]. In mice, the higher levels of V_2_R translate to a greater sensitivity: treatment with the same dose of desmopressin, a synthetic vasopressin agonist, reduces urine production and increases urine osmolarity to a greater extent in females than in males [125]. A similar difference has been reported for humans [127, 128], in which desmopressin treatment of nocturia, an abnormal urge to urinate during the night, was more likely to cause hyponatremia (abnormally low sodium concentration in the blood, usually due to increased water retention) in women than in men. Based on these findings, the authors suggested that women should be treated with half to one fourth of the dose used for men [128], although this suggestion has not been adopted.

Under normal conditions, the sex difference in kidney V_2_R expression does not appear to cause major physiological differences, but if the system is stressed, differences in osmoregulation occur that can affect the brain. For example, female marathon runners and endurance swimmers have a higher risk of cerebral edema related to hypoosmolarity than do male runners [129, 130]. This appears to be related to an increase in vasopressin release during the run [131], which may provoke a stronger kidney response in women than in men, increasing their risk for acute hyponatremia. In addition to causing differences in brain pathology, sex differences in V_2_R expression may also be related to sexual differentiation of the hypothalamic neurons that produce vasopressin. For example, the supraoptic nucleus is bigger and has larger vasopressin neurons in male than in female rats [132] and also produces more vasopressin mRNA [133]. These differences are suggested to relate to sex differences in overall body size [132], but it is possible that they are, at least in part, the result of a compensatory response to the lower levels of kidney V_2_R expression in males.

#### Sensory systems

Even given the exact same environment, males and females may experience that environment differently, due to sex differences in sensory systems. Taste, pain/touch, vision, and olfactory sensitivities differ between males and females, and there is evidence for both acute and programming effects of gonadal steroids on these sex differences.

For example, rats show sex differences in behavioral taste sensitivity to sodium chloride solutions, and sex differences in gustatory processing of the same-strength salt solution can be measured in electrophysiological recordings of the chorda tympani nerve [134]. In humans, women have a higher density of fungiform papillae on the tongue than do men [135], and differences in taste sensitivity have been reported between the sexes as well as across the menstrual cycle in women [136].

Over 40 years ago, estrogens were shown to increase the size of the pudendal nerve receptive field and the sensitivity of cutaneous receptors in the perineum of rats, making female rats more sensitive to stimulation around the vagina at the time of estrus [137]. Touch sensitivity also varies in women during the menstrual cycle in regions one would not think of as sexual, such as the cornea [138]. Pain thresholds [139] and response to common analgesics [140] vary by sex in both humans and in animal models, as does degree of pain experienced in a number of clinical conditions. For example, in a mouse model of multiple sclerosis, only females exhibit neuropathic pain (sensitivity to mechanical, cold, and heat stimulation), even when motor dysfunction does not differ between the sexes [141]. Similarly, women with osteoarthritis have significantly more pain than men, even when controlling for variables such as depression, anxiety, pain catastrophizing, social support, and physical activity [142]. Whether this is due to sex differences in the periphery or in neural processing is not known.

Sex differences in olfactory processing have received more attention than those in other sensory modalities, perhaps because the most common experimental animals (mice and rats) are highly olfactory. Well-established sex differences are found in the detection and response to socially relevant urinary odors in mice. Some sex differences persist even if all animals are gonadectomized [143], or gonadectomized and treated with an estrogen [144], indicating programming/organizational effects of hormones, or possibly a direct sex chromosomal effect, on the processing of olfactory cues. Using expression of the immediate early gene cFos as a measure of neuronal activity, a sex difference in the neural response to an olfactory stimulus can be seen as early as the vomeronasal epithelium (i.e., the primary sensory neuron detecting pheromones) [145]. While the authors considered the possibility that this sex difference is due to centrifugal inputs from the central nervous system to vomeronasal neurons [145], a recent paper pushes the envelope by showing that in the female mouse, vomeronasal sensory neurons respond to male-specific urinary proteins in estrus, but not in the diestrus phase of the cycle [146]. Because the response of the sensory detectors was studied after they were removed from the nose and dissociated in culture, centrifugal influences should have been absent. The finding was, moreover, traced to the ability of high levels of progesterone (characteristic of diestrus) to block the functioning of vomeronasal neurons that respond to male urinary proteins, while not affecting those that respond to a predator odor [146].

Not all sex differences in sensory systems are due to hormones. Sex differences in color discrimination have been linked to genetic sex because genes for retinal photopigments reside on the X chromosome. Owing to a double dose of X chromosome genes, some women have up to four different X-linked photo color pigment alleles, and these women perceive an increased number of distinct colors [147, 148]. Conversely, by having only a single set of X chromosomal alleles, males are much more sensitive to deleterious mutations in photopigment genes and have a higher incidence of color blindness [149]. Thus, gonadal steroids and sex chromosomes can alter the reception of primary sensory cues, literally changing the perceived world.

### A “Sexorganome”?

The idea that sex differences in biological systems are interdependent was recently proposed for networks of genes by Arnold and Lusis, who introduced the term “the sexome” [150]. They point out that the function of every cell in the body is the product of an intricate network of interactions among all the different molecules that make up a cell and define the sexome as “the sum of all sex-specific and sex-biased modulatory interactions that operate within [these] network[s]” [150]. Although individual sex differences in gene expression may be small, in the aggregate, these differences may importantly affect function or may prevent sex differences in other cases [150].

The idea of the sexome likely applies to every tissue in the body. As alluded to above, transcriptome studies show pervasive sex differences in gene expression in peripheral tissues. The factors that induce sex bias in gene networks are the same as those that induce sex bias in neural networks and behavior, i.e., programming and acute effects of gonadal steroids, sex chromosomes, and the environment. These factors probably affect some nodes in the network (e.g., genes with estrogen-response elements in the promoter region) more than others. As these genes form part of a network, however, changes in the expression of one gene will affect the expression of all others in the network.

**Fig. 1:**
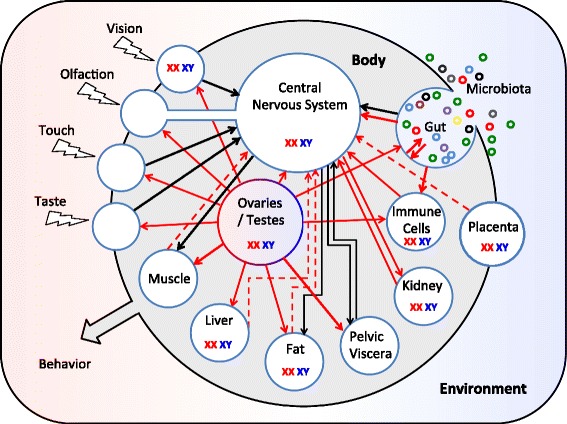
Sex differences in peripheral influences on the central nervous system. The CNS is embedded in a sexually differentiated body that is embedded in an environment, which may interact with the body in a manner that varies by sex . This diagram represents interactions between the central nervous system and sex differences elsewhere in the body that are discussed in this paper; other interactions undoubtedly occur. *Solid arrows* indicate a sex influence from one organ on another. *Dashed arrows* indicate an influence inferred from circumstantial evidence, but not yet demonstrated. *Black arrows* indicate neural communication; *red arrows* indicate humoral communication. “*XX XY*” indicates organs in which sex chromosome complement has a demonstrated effect; in most cases, it is not known whether the effect is mediated within that organ or indirectly via effects on other organs. The *small colored circles* in the *upper right* are the many species of microorganisms living commensally in our gut or on our skin

Although the sexome was originally formulated to explain the working of gene networks, it may be possible to take a more “macro” view of the sexome, where nodes in the networks are organs rather than genes. For example, sex differences in the function of the liver will affect the composition of the blood, and sex differences in the composition of the blood may affect the function of all other tissues in the body, including the brain. Just as the sexome proposed by Arnold and Lusis is the aggregate of gene expression differences, bodies are aggregates of organs influencing each other via functional ties, which could be thought of as the “sexorganome” (Fig. 1). And just as sex affects some genes more than others, the factor “sex” will affect some organs in more obvious ways than others. Figure 1 is by no means encyclopedic and merely represents some of the interactions between the brain and organs discussed in this paper, leaving out most interactions between those organs. We would be surprised if, once the data are in, there would be any organ left that could not be added to the figure.

This perspective may be useful in thinking about the implications of a sex difference, wherever in the body. Up to now, we have glossed over the fact that there is a huge variation in the magnitude of sex differences depending on brain region, organ, tissue, etc. For some measures, there is very little overlap between the sexes (such as number of SNB motoneurons or size of the projection from the BNST to AVPV discussed above), whereas for other differences the overlap between the sexes is great (such as the size of the supraoptic nucleus, discussed above as well). In addition, the magnitude of a given sex difference is often context-dependent and varies depending on age, the presence of physical or emotional stressors during development and in adulthood, etc. [15]. In seasonally reproducing species, time of year may be a factor. To the extent that organs influence each other, the effects of factors on the size of sex differences in one organ will reverberate in the form and function of other organs.

### Beyond the body

Although most of the interactions described above take place within a single body, there are also more circuitous routes by which sex can affect the brain and behavior. We give just a few examples here.

#### Placenta

The placenta is comprised of cells derived from both the mother and the fetus and is therefore sex-specific (only male fetuses have a placenta containing male cells). Maternal diet affects gene expression in the placenta and does so differently depending on whether the placenta is male or female [151]. There are also sex-specific responses of the placenta to stressors [152, 153], which would be expected to influence how male and female fetuses experience perturbations in utero, and may affect later brain function. When pregnant female rats were subjected to a stressor during early gestation, male but not female offspring exhibited adverse effects on stress responsivity, anhedonia, and response to selective serotonin reuptake inhibitors in adulthood [154]. The placenta appears to be the site of action for these sex-specific responses because the early prenatal stress causes elevations of immune response genes, including interleukin-6 and interleukin-1β, specifically in male placentas, and the stress phenotype in males could be blunted by blocking this response [155]. Here is a scenario, then, where a stressor experienced by one animal (the mother) influences brain development of the offspring in a sex-dependent manner and does so via effects on a tissue that is outside of the “body proper” and neither entirely fetal nor maternal!

#### Muscles and teeth

Overall, muscle mass is much larger in males of many mammalian species. In humans, which are less dimorphic than many other primates, total skeletal muscle mass is about 60 % greater in men than in women [156]. As far as we know, this is not normally accompanied by differences in motoneuron number but could affect the brain and behavior more indirectly. Canine tooth size is also up to 400 % larger in the males of some anthropoid primates [157], and this difference is at least partially due to prenatal androgens [158, 159]. It is not hard to imagine that endowing an animal with greater overall size, greater muscle mass, and fourfold larger canines might affect its behavior. Whether it is feedback the individual gets about his/her own strength or the reaction of other individuals, the brain will not fail to notice. Currently, however, we really do not know where in the brain to look for such effects.

Because complicated questions sometimes yield to simpler model systems, worms may be useful here. William Mowrey, Douglas Portman, and colleagues recently conducted the worm equivalent of the thought experiment proposed above. They examined locomotor behavior in male *Caenorhabditis elegans* roundworms with a “female” nervous system and “female” worms with a male nervous system (female is in quotations here because although *C. elegans* come in two sexes, they are males and hermaphrodites—modified females capable of self-fertilization). Sexual dimorphism in *C. elegans* is cell autonomous and depends on expression of the *tra-1* gene [160]. Thus, by expressing or repressing *tra-1* in specific cell types, one can mix and match the sex of various tissues within the same worm.

Locomotion in male and hermaphrodite *C. elegans* involves sine-wave-like body undulations that differ along several dimensions. By examining locomotion in male worms with a hermaphrodite nervous system (and vice versa), one dimension (body-wave frequency) was shown to be determined completely by the sex of the nervous system. In fact, body-wave frequency could be sex-reversed by reversing only the sex of *sensory* neurons [161]. Locomotor velocity, on the other hand, was not affected by sex of the nervous system but could be masculinized in hermaphrodites with male *muscle* cells. Neural plus muscle sex reversal came close to completely sex-reversing locomotion, but something was still missing. The authors hypothesize that the missing piece is related to biomechanics, i.e., the different overall size of male and hermaphrodite bodies interacting with the substrate along which they move [161]. Thus, the “sex” of even this very basic behavior—sine wave locomotion in worms—is distributed among sensory neurons, muscles, and the interaction of the individual with the environment.

As the worm example suggests, and as researchers studying the biomechanics of locomotion have long known, if brains are wired up to different bodies, similar neural output can have different consequences [162]; conversely, to generate similar behaviors, the nervous system residing in male and female bodies may have to compensate by giving different commands. The interaction of the size and shape of the body with the medium it travels through points to the final variable we consider here—the environment.

#### The environment—“It takes a village…”

One very important indirect effect of gonadal hormones on the brain is likely mediated via effects on the genitalia which, in humans at least, change the way we are treated from the moment of birth, indeed, before birth, if parents know the sex of the fetus [163]. Later in life, being categorized as “male” or “female” may continue to have a profound influence on the social input the individual receives, with consequences for the brain.

These effects are not limited to humans. Male rats are licked more frequently by their mothers than are females. Variations in maternal licking affect later sexual behavior [164], the number and morphology of motoneurons in the SNB [165, 166], and several measures in the brain (for example, [167, 168]). Differential treatment of male and female pups by the mother is caused by differences in the urine, due to male pheromones produced under influence of testosterone by the preputial glands [169]. Therefore, the chain of events seems to be that sex chromosomes cause differences in the gonads, which cause differences in testosterone production, causing a different constitution of the urine, which affects the behavior of another individual (the mother), and thereby the developing nervous system and behavior of the individual. Somatosensory contact by the mother also affects juvenile play behavior in her offspring and does so in a sex-dependent manner [170]. In turn, juvenile play alters social and sexual interactions in adulthood, suggesting another complex cascade of effects. To make matters more interesting, interactions are not limited to those with conspecifics—as mentioned above, sex differences in the microbiota, for example, indicate that our treatment by, or “attractiveness” to, other species also varies by sex.

One justified criticism of our field has been that results are often presented as though sex is hardwired, that having a Y chromosome, and therefore testes, sets in motion a process towards maleness that is unavoidable. Well, it depends on how you look at it. On the one hand, different treatment from others (whether “others” are caregivers or bacteria) is presumably a response to some physical or behavioral sex difference(s) (genitalia, physique, gait, activity level, biochemistry of intestines, chemicals in urine, to name a few), which is a manifestation of programming and acute effects of hormones and differential expression of sex chromosomal genes. In other words, the impetus for sexual differentiation is the Y chromosome and differences in sex steroids, but the routes via which these factors exert their effects are more circuitous than is (often tacitly) implied in the literature. For humans, with extensive social interactions and long development times, this means that there are plenty of opportunities to override or, alternatively, magnify the initial “program.”

## Conclusion

### Back to the future

In the early days, Frank Beach famously resisted the conclusion that differences in sexual behavior in male and female rodents were caused by differences in the brain, instead pointing to feedback from the genitalia as an obvious possible mediator (for discussion, see [171]). Although later research, including that by Beach himself [172], proved that one can masculinize behavior without corresponding changes to the genitalia [173, 174], the early Beach was on to something. The realization that hormones have direct actions on the brain was exciting, and in our embrace of this idea, the pendulum may have swung too far. Over the last 50 years, research in the field of neural sex differences has only rarely considered a role for other organs. With a single-minded focus on the brain, this organ may appear to have special access to the whisperings of sex chromosomes or sex steroids. All organs are sexually differentiated to some degree, and keeping the whole body in mind opens the aperture to consider novel mechanisms and pathways for the development of normal sex differences, as well as the mechanisms underlying neurological disorders and diseases that show sex differences in onset, course of disease, and morbidity.

## Abbreviations

AVPV: Anteroventral periventricular nucleus of the hypothalamus
BNST: Bed nucleus of the stria terminalis
CNS: Central nervous system
MS: Multiple sclerosis
SNB: Spinal nucleus of the bulbocavernosus
V_2_R: Vasopressin V_2_ receptor
